# Montana Statewide Google Earth Engine-Based Wildfire Hazardous Particulate (PM2.5) Concentration Estimation

**DOI:** 10.3390/air2020009

**Published:** 2024-05-02

**Authors:** Aspen Morgan, Jeremy Crowley, Raja M. Nagisetty

**Affiliations:** 1 Autonomous Aerial Systems Office, University of Montana, Missoula, MT 59812, USA; 2 Department of Environmental Engineering, Montana Technological University, Butte, MT 59701, USA

**Keywords:** Google Earth Engine, PM2.5, random forest, predictive modeling

## Abstract

Wildfires pose a direct threat to the property, life, and well-being of the population of Montana, USA, and indirectly to their health through hazardous smoke and gases emitted into the atmosphere. Studies have shown that elevated levels of particulate matter cause impacts to human health ranging from early death, to neurological and immune diseases, to cancer. Although there is currently a network of ground-based air quality sensors (*n* = 20) in Montana, the geographically sparse network has large gaps and lacks the ability to make accurate predictions for air quality in many areas of the state. Using the random forest method, a predictive model was developed in the Google Earth Engine (GEE) environment to estimate PM2.5 concentrations using satellite-based aerosol optical depth (AOD), dewpoint temperature (DPT), relative humidity (RH), wind speed (WIND), wind direction (WDIR), pressure (PRES), and planetary-boundary-layer height (PBLH). The validity of the prediction model was evaluated using 10-fold cross validation with a R^2^ value of 0.572 and RMSE of 9.98 μg/m^3^. The corresponding R^2^ and RMSE values for ‘held-out data’ were 0.487 and 10.53 μg/m^3^. Using the validated prediction model, daily PM2.5 concentration maps (1 km-resolution) were estimated from 2012 to 2023 for the state of Montana. These concentration maps are accessible via an application developed using GEE. The product provides valuable insights into spatiotemporal trends of PM2.5 concentrations, which will be useful for communities to take appropriate mitigation strategies and minimize hazardous PM2.5 exposure.

## 1. Introduction

Wildfires pose a direct threat to the property, life, and well-being of the population of Montana, and indirectly to their health through hazardous smoke and gases emitted to the atmosphere, such as CO, CO_2_, and CH_4_ [1]. Wildfires in Montana are primarily caused unintentionally by human error [2] or are caused naturally by cloud-to-ground lightning strikes [3]. Regardless of the causes of wildfires, studies have shown that elevated levels of particulate matter cause impacts to human health ranging from early death to neurological and immune diseases to cancer [4]. The Global State of Air from 2019 details that almost 5 million people (about twice the population of Mississippi) die prematurely annually from exposure to outdoor air pollution [5,6]. Fine particulate matter 2.5 (PM2.5) is a standard EPA measure that describes fine inhalable particles that are 2.5 μm (or microns) in diameter or smaller [7]. The risk to those living in urban areas is even greater due to anthropogenic emissions from transportation, industry, and energy production [8,9]. In the past forty years, wildfire burn areas have quadrupled in the US [10]. One study finds that wildfires contribute to 25% of PM2.5 in the US, with some western regions having up to 50% contribution of PM2.5 from wildfires [11].

**Figure 1.**
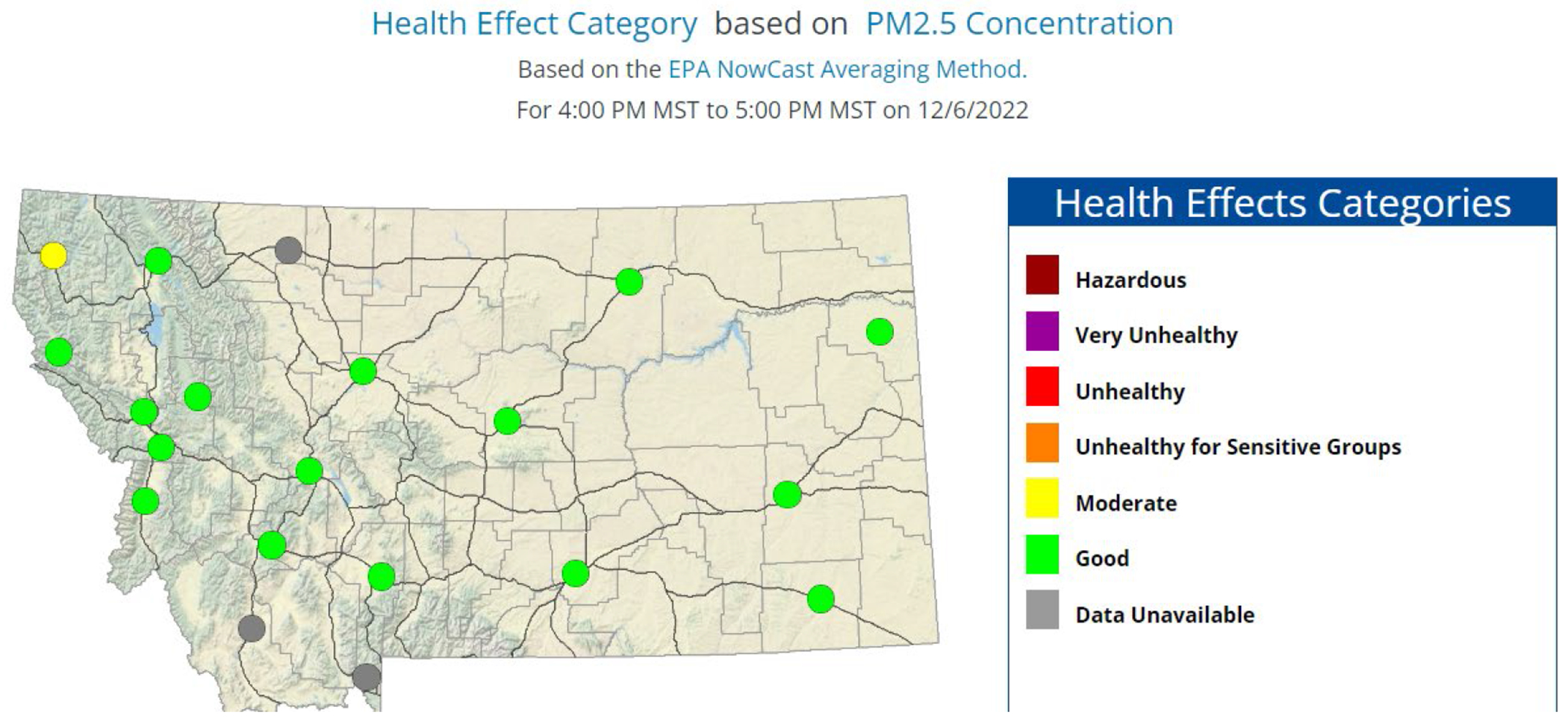
MT DEQ webpage screenshot showing the DEQ ground-based stations for tracking PM2.5 in Montana [12].

Although there is currently a network of ground-based air quality sensors (20 stations listed in the Montana Department of Environmental Quality’s (DEQ) Air Quality and Smoke Program) in Montana (Figure 1), the geographically sparse network has large gaps and lacks the ability to make accurate predictions for air quality in many areas of the state [12]. The DEQ Air Quality and Smoke program is based on the US Environmental Protection Agency (EPA) Nowcast system for determining the Air Quality Index, which uses a time-average calculation based on hourly particulate matter data.

Montana has a ground-based air quality system in place to warn residents of the potential for hazardous air conditions, but the sparsity of monitoring locations leads to uncertainty in many rural areas of the state. There is also a lack of prediction capacity in this monitoring system, since it is based on current conditions at the monitoring stations.

### 1.1. PM2.5 Prediction Using Satellite-Based Data

Over the past five decades, satellite systems have evolved to deliver higher resolution, to have faster return times, and to have sensors to analyze more specialized variables for earth observation. For example, the Sentinel 5P satellite can detect several trace gasses and aerosols in the atmosphere at high spatial resolution [13]. Advances in cloud storage and processing and atmospheric modeling have also improved significantly over the past decade, including the creation of the Google Earth Engine platform in 2010 [14]. These advances in satellite spatial resolution, cloud computing, and atmospheric modeling can vastly improve our predictive abilities for hazardous air quality. Aerosol optical depth (AOD) is defined as the measure of aerosols (i.e., smoke particles, sea salt, desert dust) within a column of air from the earth to the top of the atmosphere [15]. The MODIS Terra and Aqua Land Aerosol Depth product data are produced daily at a 1 km resolution. The MODIS Terra (also known as EOS AM-1) and Aqua (also known as EOS PM-1) satellites are timed so Terra passes from north to south across the equator in the morning and Aqua passes the equator in the afternoon. The combination of the two satellites covers the entire Earth surface every 1–2 days [16].

Researchers have used simple linear and multiple linear regression and machine learning methods to predict PM2.5 based on AOD, land use, and meteorological data (Table 1). Wang and Christopher [17] correlated MODIS AOD with PM2.5 using simple linear regression and reported a linear correlation coefficient of 0.7. More sophisticated statistical models have also been used to predict PM2.5. For example, Kloog et al. [18] used a mixed effects model to predict PM2.5 using land use and meteorological data. Among these more sophisticated models, ensemble machine learning models have been among the most popular for predicting PM2.5 using satellite data. Ensemble models combine smaller models to generally reduce overfitting and improve results. Random forest and gradient boosting are the most common ensemble models applied to this problem.

The random forest method can model non-linear relationships between independent and dependent variables. Random forest regression is built on a simpler model called a decision tree. Decision trees are highly sensitive to changes in data and are more effective when combined. Random forest regression predicts values using the mean output of decision trees that are each trained on different variations of the data. The variations in the training data are created using bootstrapping, which is a statistical method of sampling a dataset with replacement to create pseudo-new datasets.

### 1.2. Objectives of the Study

The goal of the study was to develop a method to estimate daily PM2.5 concentrations for Montana using daily satellite data. The objectives of the study were twofold: first, to develop a random forest regression model to predict PM2.5 concentrations using satellite-based measurements. Second, to use a random forest model to convert daily satellite data into a statewide daily PM2.5 concentration map. The study significantly contributes to the available PM2.5 concentration data, allowing individuals to take measures to mitigate exposure during the wildfire season.

## 2. Study Area

Montana is the fourth largest state in the US, with a total area of 380,800 km^2^ [24]. The total population of Montana is 1,122,867, and it is ranked 43rd in total population and 48th in population density (2.73/km^2^) [25] in the US. Forests cover about 25% of Montana, totaling about 22.5 million acres (9.1 million hectares), dominated by firs, larch, lodgepole and hemlock [26]. About 33% of Montana is public lands (state and federal) totaling over 30 million acres (12.14 million hectares) [27].

Wildfires are very common in Montana, and pose a significant threat to people, long-term health, and property. Montana’s peak fire season is typically July through September, and in 2023 there were 501 reported fire alerts. The most fires recorded in a year in Montana was in 2017, with 4418 reported fires [28]. According to the National Interagency Fire Center (NIFC), over the past decade there was an average of 61,376 wildfires per year and an average of 7.2 million acres (about the area of Massachusetts) burned per year [10] in the US.

During the 2020 fire season, western wildfires contributed 42% to surface PM2.5 measurements in the mountain region and wildfires were the primary contributor to 3720 exceedances of the National Ambient Air Quality Standard for PM2.5. During the peak days, wildfire contribution to surface PM2.5 reached 72% in the inter-mountain region [29].

The average summer-long surge in PM2.5 due to fires is 1.84 μg/m^3^ in Forest Service Region 1 (which includes Montana) and is approximately doubled during large-fire years [30]. Since 1950, the western US has seen near exponential growth in fire frequency and size, as well as the increased occurrence of megafires (burns of more than 100,000 acres) [31]. Estimates of future fire-related PM2.5 increases range from 55% to 190% and the number of premature deaths in the continental US is expected to double by 2100 [32].

## 3. Methods

### 3.1. Data Sources

#### 3.1.1. PM 2.5 Concentrations

**Figure 2.**
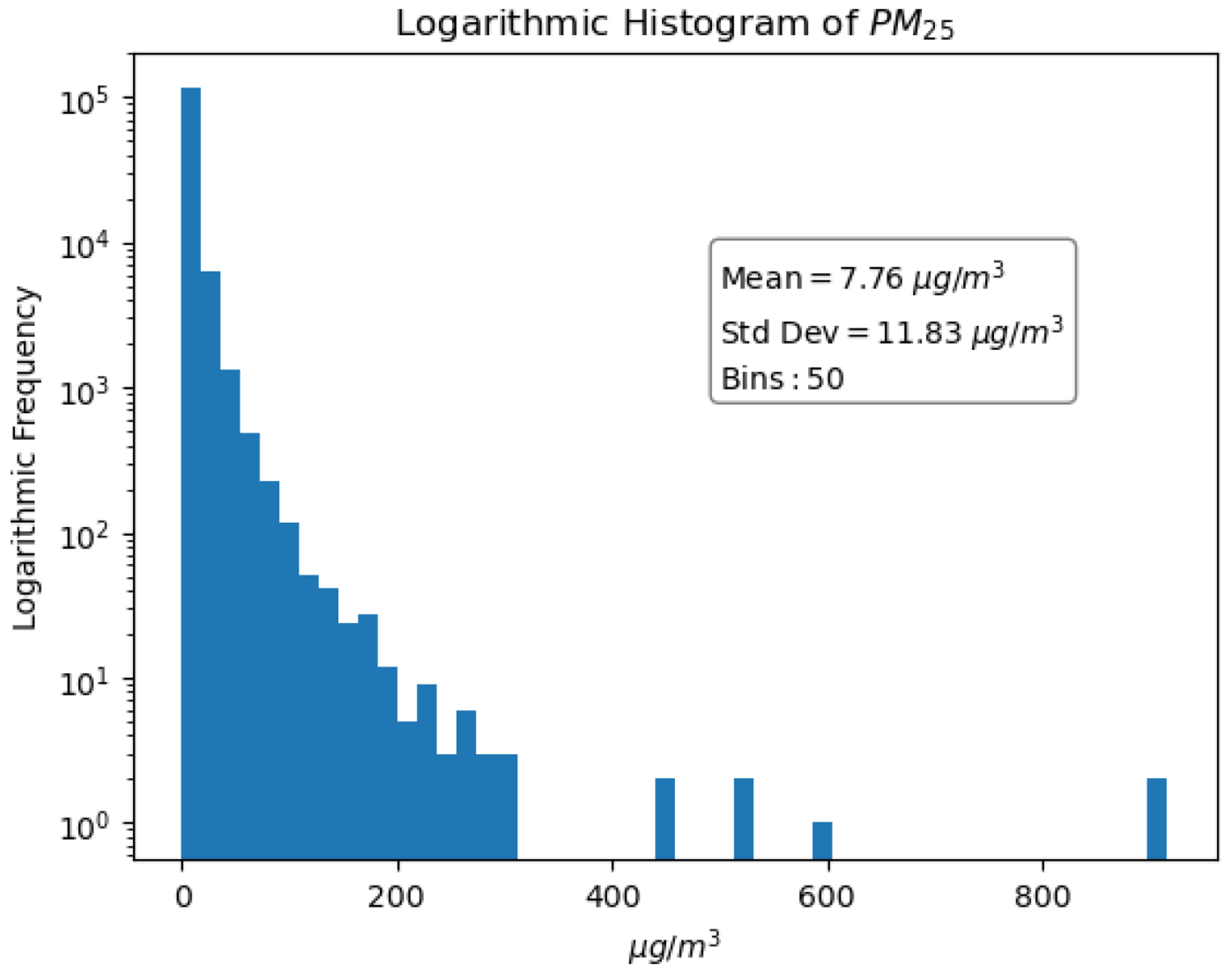
Logarithmic distribution of PM2.5 data.

**Figure A1.**
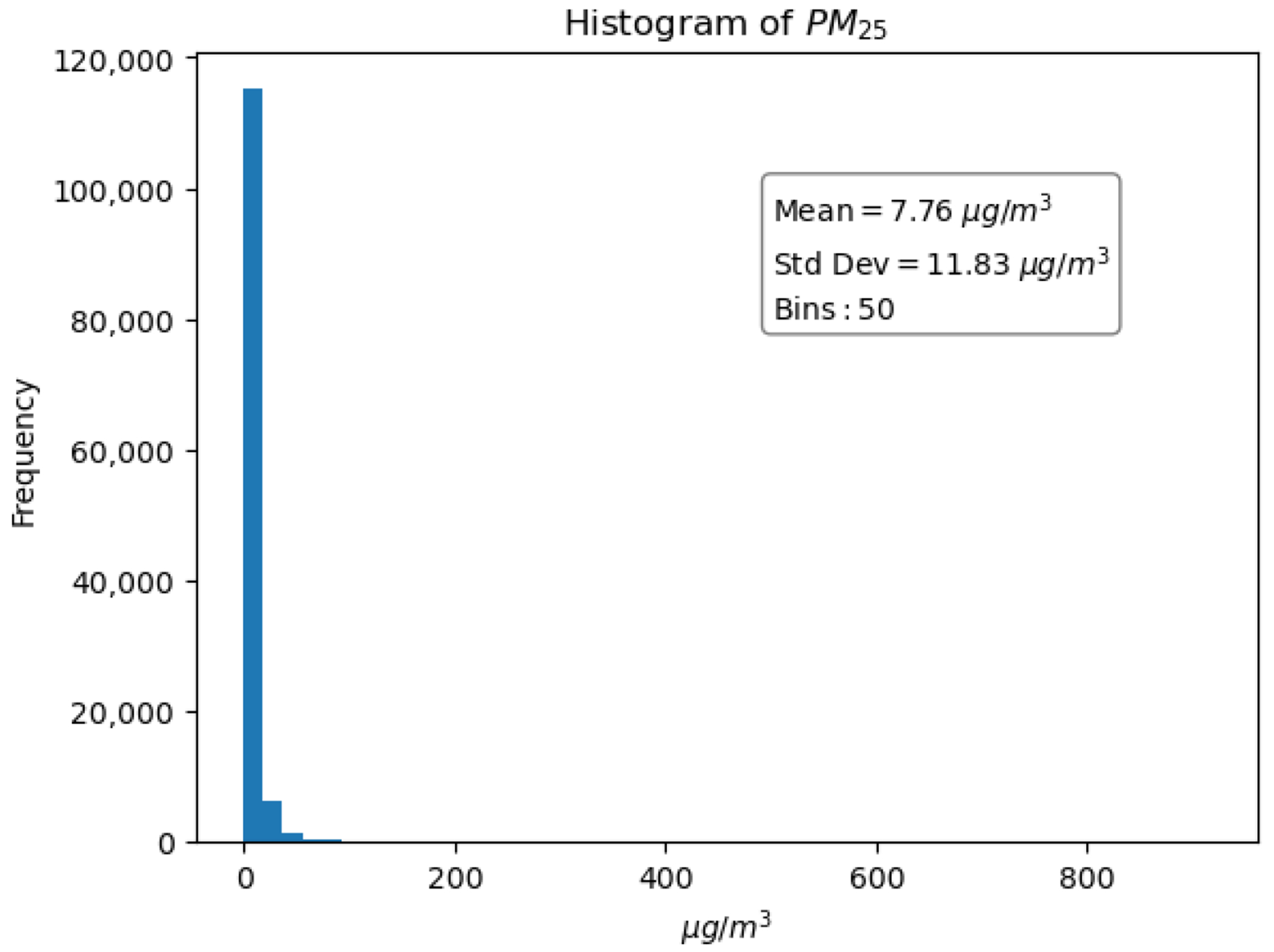
Distribution of PM2.5 before integrating data.

PM2.5 concentration data for Montana was downloaded from the Montana Department of Environmental Quality webpage (Table 2, [33]). The ground-station data was averaged over the 17:00 to 19:00UTC and 19:00 to 21:00UTC time-windows and then formatted into a Google Earth Engine (GEE) tabular data structure. There are missing and faulty observations (negative values and values exceeding 1000 μg/m^3^) from the 20 ground stations that measure PM2.5 hourly. Faulty PM2.5 values were removed prior to data integration. Of the 176,660 expected observations (two per day per station over 10 years), there were 123,932 observations left, due to missing or invalid values. The distribution of the 123,932 observations is available in Figure 2. The distribution without logarithmic scaling is available in the (Figure A1).

#### 3.1.2. Google Earth Engine Cloud Platform

The GEE platform combines a huge catalog of geospatial datasets with the GIS and statistical tools sufficient to analyze them [35]. The GEE platform offers JavaScript and Python Application Programming Interfaces (APIs). The GEE Code Editor runs JavaScript and allows users to visualize GEE objects easily, including a built-in map for viewing geospatial data and the incorporation of Google Charts. The GEE Code Editor can also be used to create dynamic GEE Apps for sharing results. Both APIs include advanced statistical tools, including several machine learning models such as random forest, gradient tree boosting, k-nearest neighbors, support vector machine, and naive Bayes.

#### 3.1.3. MCD19A2 AOD

GEE provides the MCD19A2 dataset, which includes AOD from Terra and Aqua satellites at 1km using the multi-angle implementation of atmospheric correction (MAIAC) algorithm [36]. The Terra and Aqua satellites each cover Montana twice daily. In this study, we used two time-windows to extract 470 nm wavelength AOD values each day. The Terra satellite completes a midday pass over Montana between approximately 17:00Z and 19:00Z and the Aqua satellite completes a midday pass over Montana between approximately 19:00Z and 21:00Z [37]. The MCD19A2 dataset guide recommends cloud masking based on given quality-control bands. Although cloud masking reduces available data, it is not the primary reason that AOD data is missing. Seventy-five percent of the AOD data is missing prior to cloud masking, and cloud masking removes an additional 10 percent of possible data points (Table 3). The majority of missing MCD19A2 AOD is likely from snow reflectance or additional heavy cloud cover. Cloud masking improved the results of both the linear-regression and random-forest models and was used for all models (Table 4).

**Figure 3.**
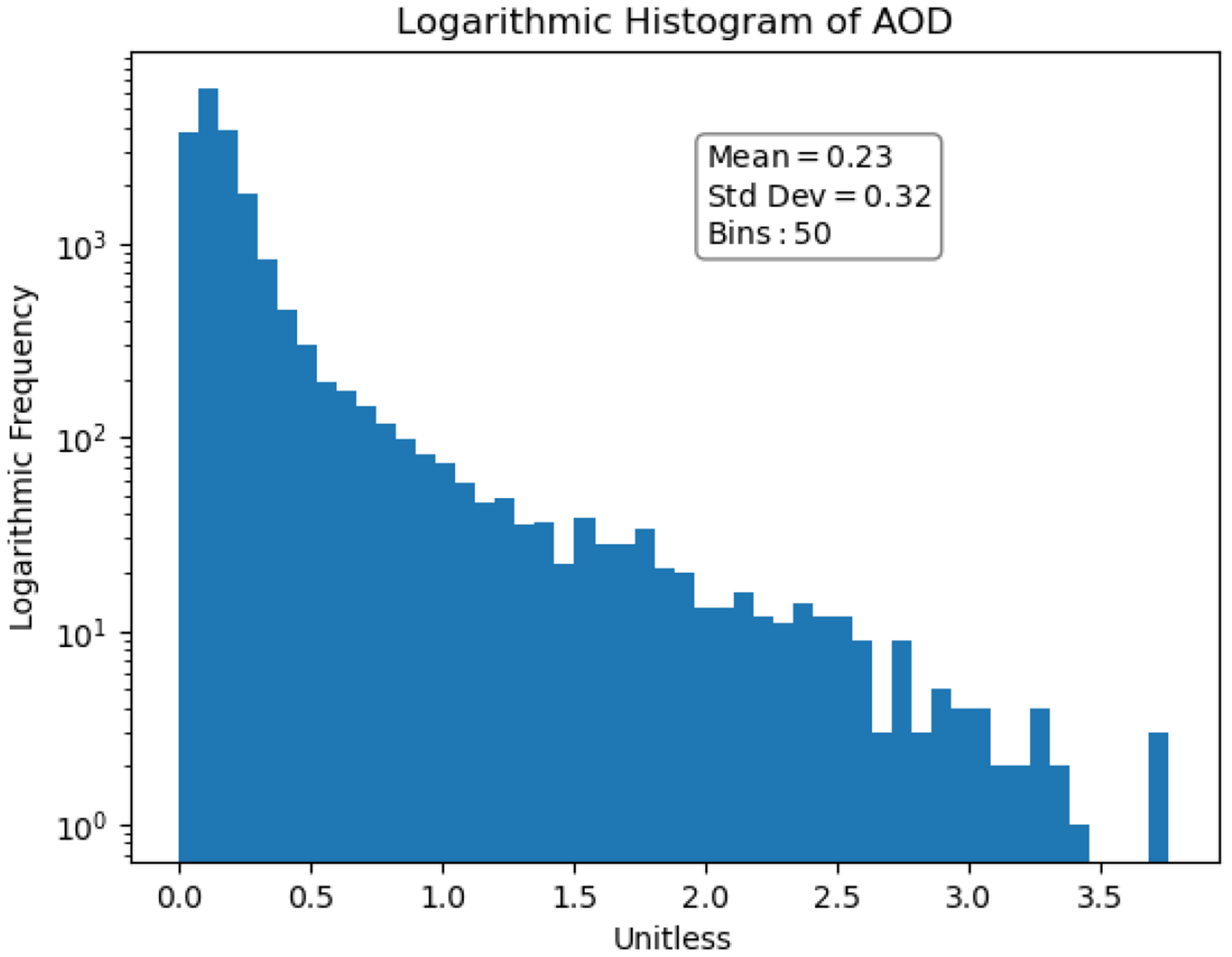
Logarithmic distribution of AOD.

**Figure 4.**
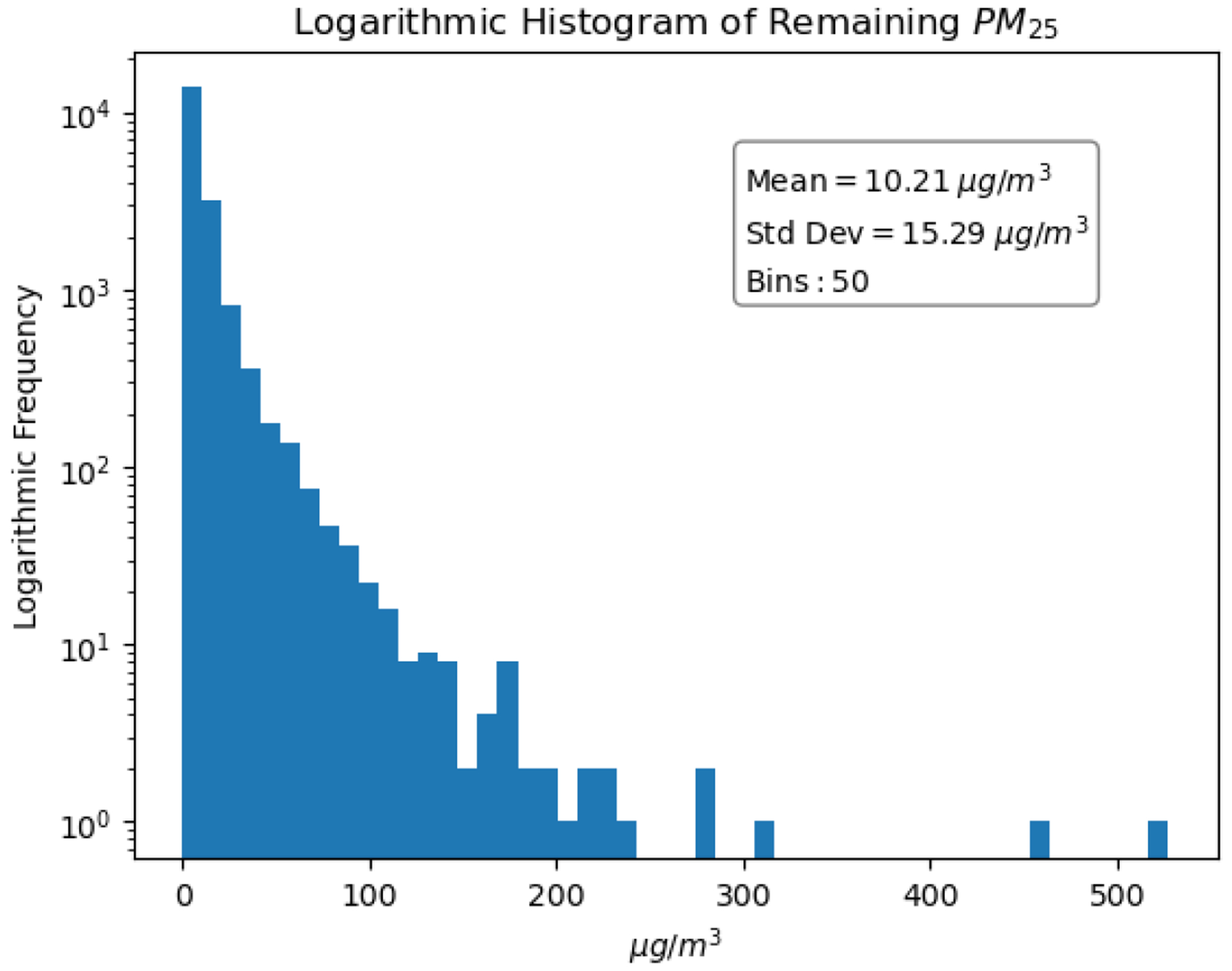
Logarithmic distribution of PM2.5 after integrating with AOD data.

**Figure A2.**
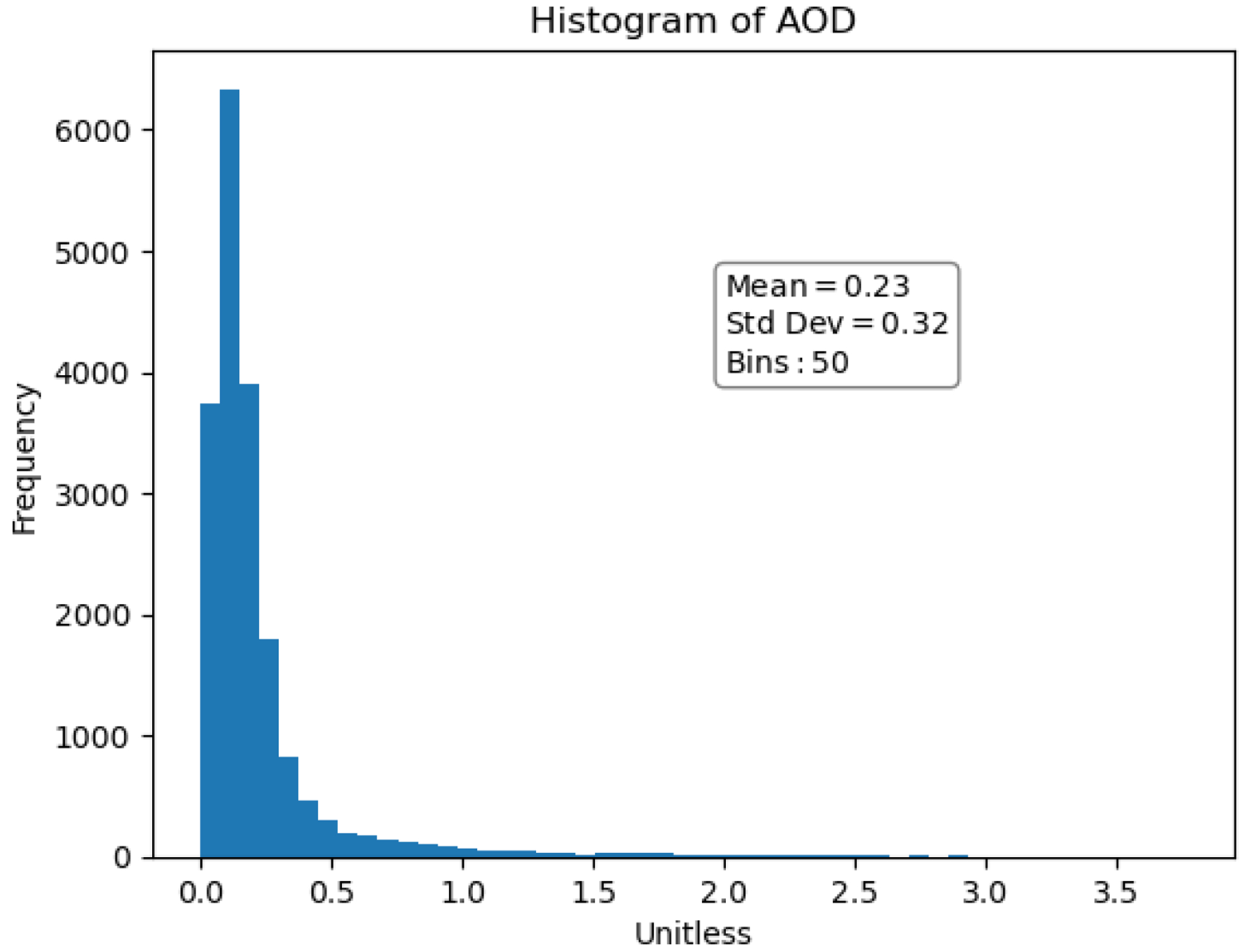
Distribution of AOD.

**Figure A3.**
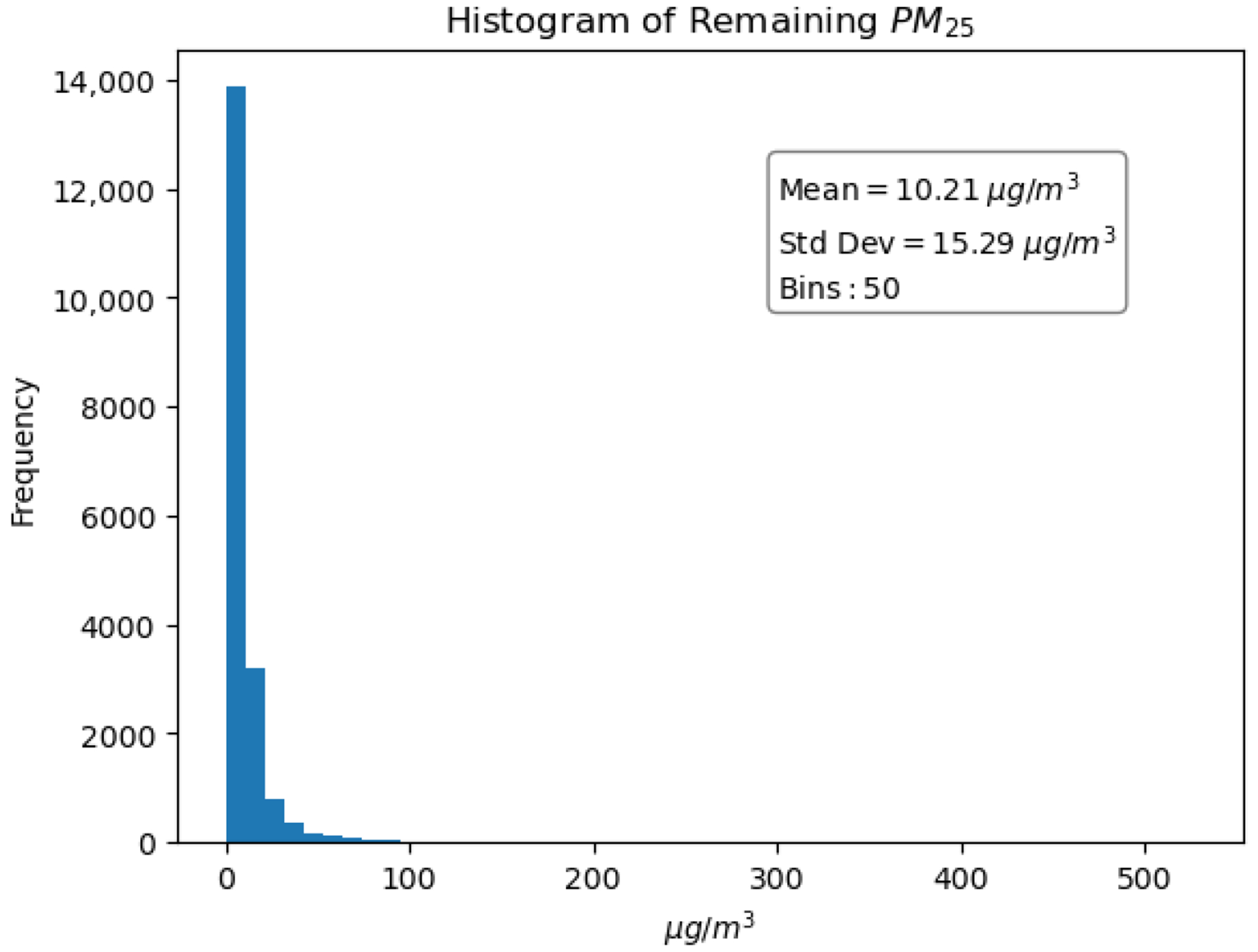
Distribution of PM2.5 after data integration.

The distribution of the cloud-masked MCD19A2 AOD data is shown in Figure 3. Although there is a positive skew to the AOD data, it is less severe than in the PM2.5 data. Since there is a significant amount of data loss in the AOD dataset, the PM2.5 distribution after co-locating with the AOD data is shown in Figure 4. Distributions without logarithmic scaling are available in the in Figures A2 and A3.

#### 3.1.4. Other Satellite-Based Weather Data Sources

In addition to aerosol optical depth (AOD), several other climate variables were introduced to improve modeling. Many studies have utilized temperature, relative humidity, pressure, wind speed, and wind direction [19,20]. Hu et al. [19] used dewpoint temperature and Brokamp et al. [20] used planetary-boundary-layer height. We used pressure (PRES), wind speed (WIND), wind direction (WDIR), and dew point temperature (DPT) from NOAA NWS RTMA [38] and planetary-boundary-layer height (PBLH) from the M2T1NXFLX data product [39]. These variables were selected based on availability on the GEE, on ability to improve model performance, and on their efficacy in related studies. Although temperature was utilized in other studies, we did not utilize it any of our models. Temperature was excluded from the linear regression models, since it is colinear with relative humidity and dewpoint and temperature was excluded from the random forest model since it did not improve modeling results. The dew point temperature, pressure, wind speed, and wind-direction data were missing for some of the available cloud-masked AOD observations (Table 3), but the relative data loss from each was insignificant.

### 3.2. Generated Tables of PM2.5 and Corresponding Satellite Data

**Figure 5.**
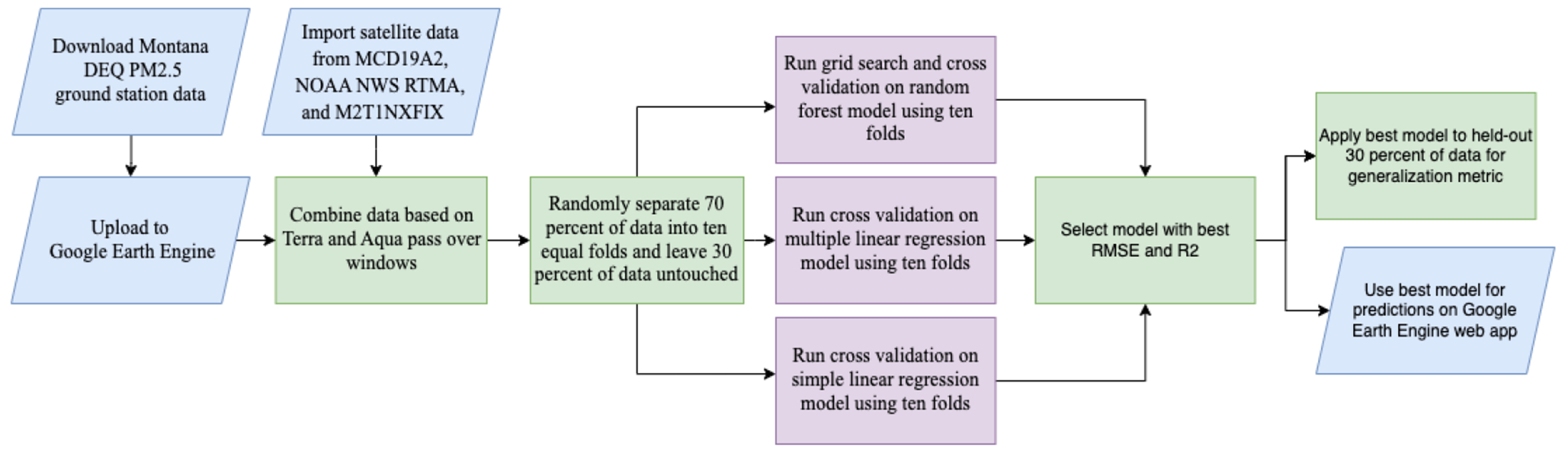
PM2.5 prediction-model-development flow chart.

To co-locate the ground-station PM2.5 and the satellite datasets (Figure 5), the average values at the ground stations during each satellite window were uploaded to GEE. Since the Montana DEQ publishes the hourly PM2.5 values at the beginning of the subsequent hour, the 17:00Z-to-19:00Z satellite window corresponded to the average of PM2.5 values from 18:00Z to 19:00Z, and similarly, the 19:00Z-to-21:00Z satellite window corresponded to the average of PM2.5 values from 20:00Z to 21:00Z. For each time window, the satellite datasets were filtered to necessary bands, composited by the mean band values over the time window, reprojected into the 1984 World Geodetic System, and scaled. The MCD19A2 Optical_Depth_047 (AOD) was cloud masked to the highest standards described in the dataset guide [36] Then, the bands in each composite image were concatenated, creating a single image containing the AOD, TMP, PRES, DPT, WIND, WDIR, and PBLH bands. The raster data were matched to the corresponding PM2.5 data at each ground-station. Null values of all variables and negative AOD values were filtered out and RH was calculated using DPT and TMP [34].

### 3.3. Predicting PM2.5 with Satellite-Based Data

Several studies have promising results from using random forest models for predicting PM2.5 from satellite data (Table 1). Random forest is an ensemble machine learning model built on simpler models called decision trees. In random forest, these decision trees are split based on a random subsample of the predictor variables and each tree is built on a bootstrapped sample of the training data [40]. Bootstrapping is the statistical method of sampling data with replacement to generate pseudo-new datasets [40].

Although random forest models are generally effective without much tuning, there are several hyperparameters that can be adjusted for optimization. These hyperparameters vary with respect to the implementation of the random forest algorithm. GEE utilizes the Statistical Machine Intelligence and Learning Engine (SMILE) library for random forest, gradient tree boosting, and certain other machine learning algorithms available in the GEE API [13]. The SMILE random forest hyperparameters selected for tuning were the bagging fraction, the minimum leaf value, and the minimum variables per split. The minimum leaf value and the variables-per-split limit the depth of individual decision trees. The leaves are the terminal nodes on the tree that output predicted values based on the data traversal through the tree [40]. The variables-per-split are the number of random predictor variables available to choose from at each split.

**Figure 6.**
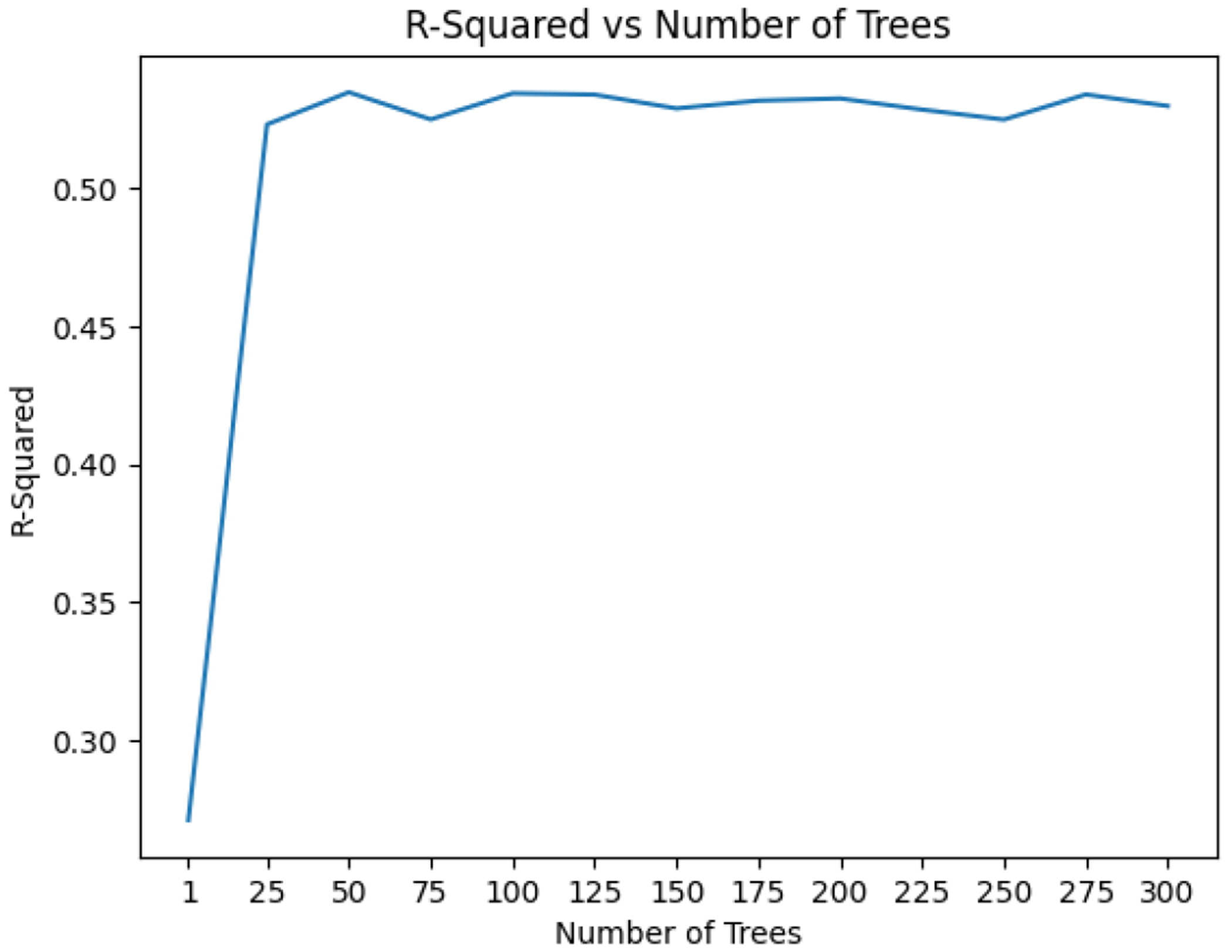
R-Squared vs number of trees using a 70–30 split in training data.

We performed a complete grid search over select hyperparameters, minimizing the root-mean-squared error. Standard scaling and normal scaling of the features decreased performance and were omitted. The predictive performance of the model based on the number of trees was evaluated with the other parameters left at default to decrease the time complexity of the grid search. Since the increase in predictive performance after 100 trees was negligible, 100 trees were used (Figure 6).

### 3.4. Cross Validation

Cross validation was utilized to compare the efficacy of the various models. Cross validation more accurately measures how well the model will perform on new data [40]. Thirty percent of the data available during the 10-year period from 2012 to 2022 was held out for a final performance test. The other 70 percent of data was randomly split into 10 approximately equal subsets (or folds). The model was trained on nine folds and tested on the remaining fold. This process was repeated ten times (per combination of hyperparameters) and a different fold was selected for testing on each iteration. The hyperparameters with the lowest cross-validated RMSE were selected for the final model (Table 5). The nine folds of data with the lowest RMSE, using the optimized hyperparameters, were selected for training the final model.

### 3.5. Montana State-Wide PM2.5 Concentration Map

The GEE JavaScript API provides the framework for publishing interactive, web-based applications for displaying data analysis performed on their platform. These applications are public interfaces for reading and visualizing results using charts and interactive maps. GEE hosts the applications on Google Cloud for free, and permits 100 requests per second among all viewers on each application. Academic organizations can request a free, one-year increase to this quota [13].

## 4. Results and Discussion

### 4.1. PM2.5 Concentrations in the State of Montana

In total, we extracted 123,932 total data points from 20 ground-based stations spread over the State of Montana from 2012 to 2022. Of these data points, 18,800 were available after matching each data point to the three satellite datasets. Each of the 18,800 data points contain PM2.5, aerosol optical depth (AOD), relative humidity (RH), wind direction (WDIR), wind speed (WIND), pressure (PRES), dewpoint temperature (DPT) and planetary-boundary-layer height (PBLH).

PM2.5 concentrations in Montana have significant spatial variation (for example, see Table 6). This is unsurprising, since Montana is the fourth-largest state in the US and has a total area of 380,800 km^2^ [24]. During 2017, residents of Seeley Lake, Montana, experienced extremely high levels of PM2.5 (average of 220.9 μg/m^3^) due to wildfire smoke from 31 July to 18 September (Table 6). Researchers discovered a significant decrease in lung function in Seeley Lake residents that remained decreased for two years following the exposure [41].

### 4.2. PM2.5 Random Forest Predictive Model

**Figure 7.**
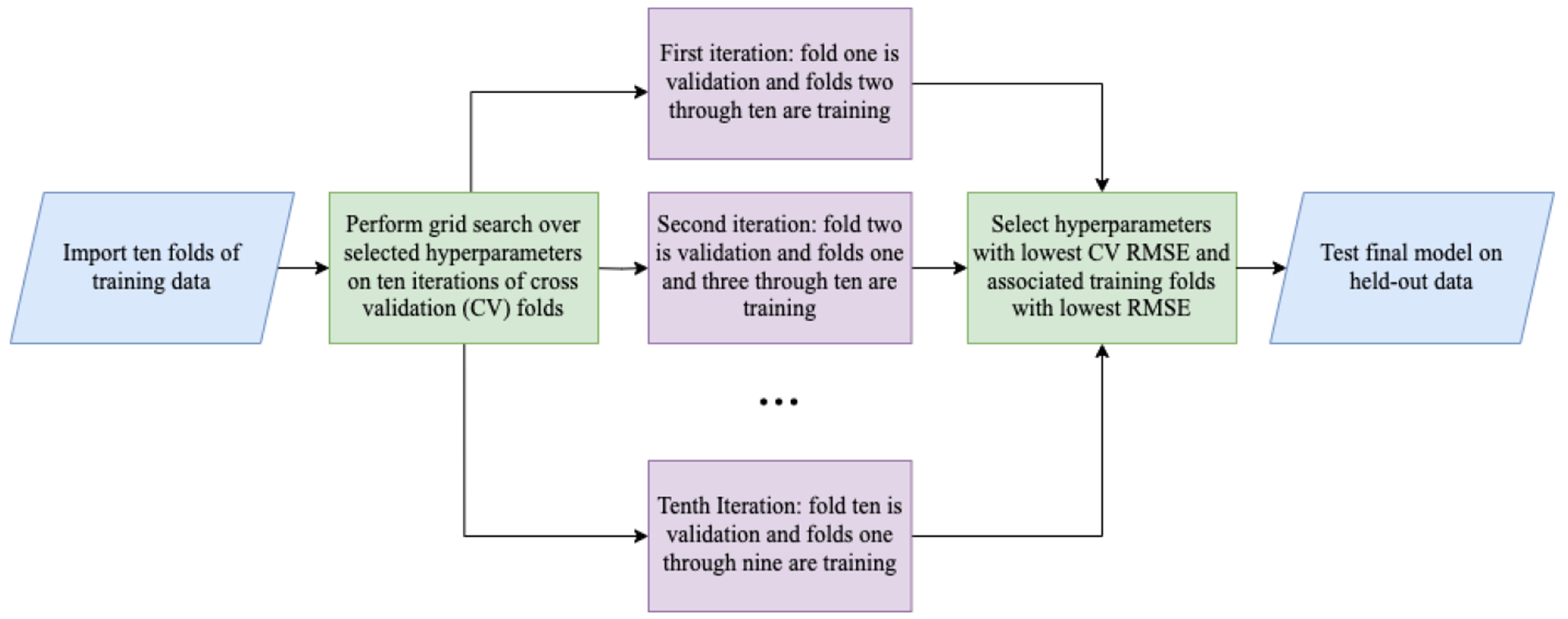
Random-forest-method cross-validation grid search.

**Figure 8.**
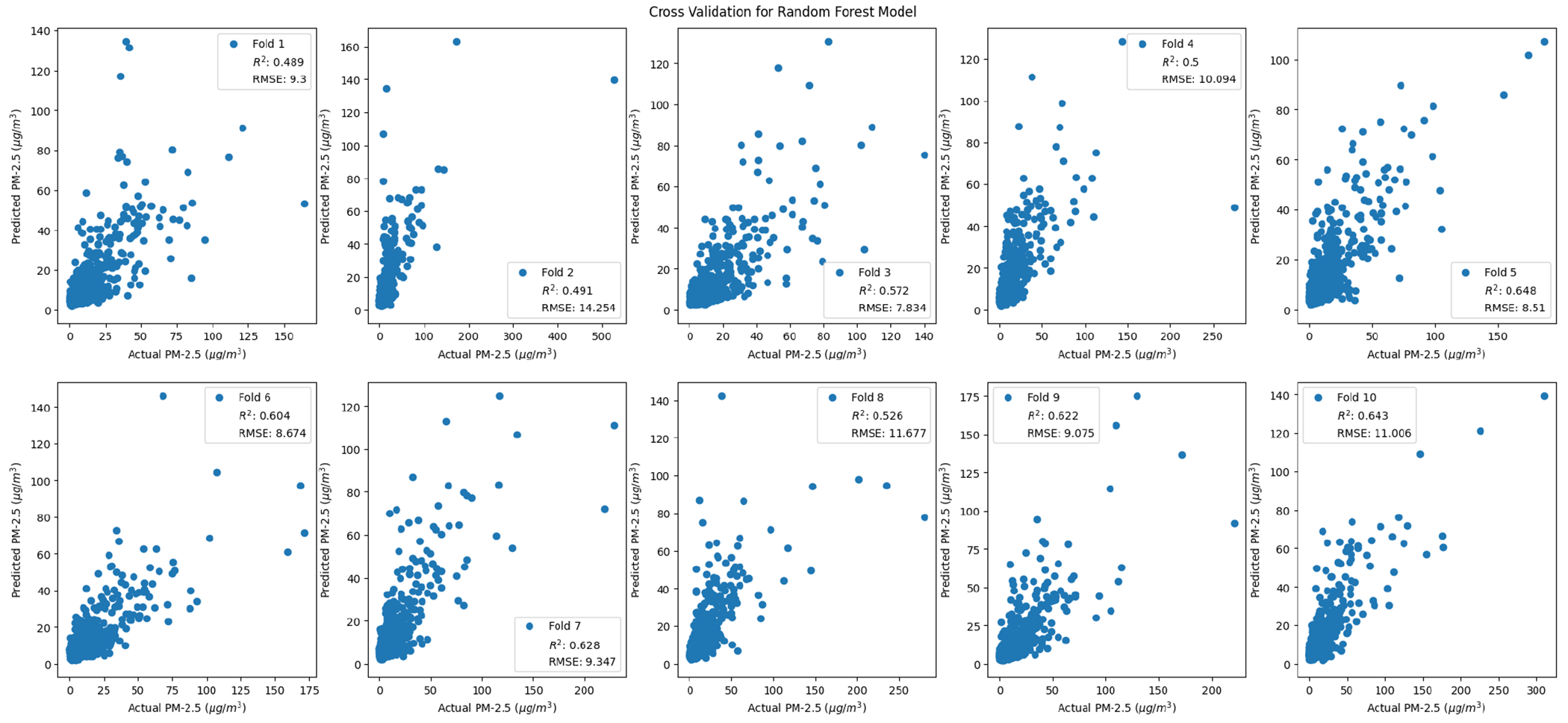
Random forest cross-validation folds.

**Figure 9.**
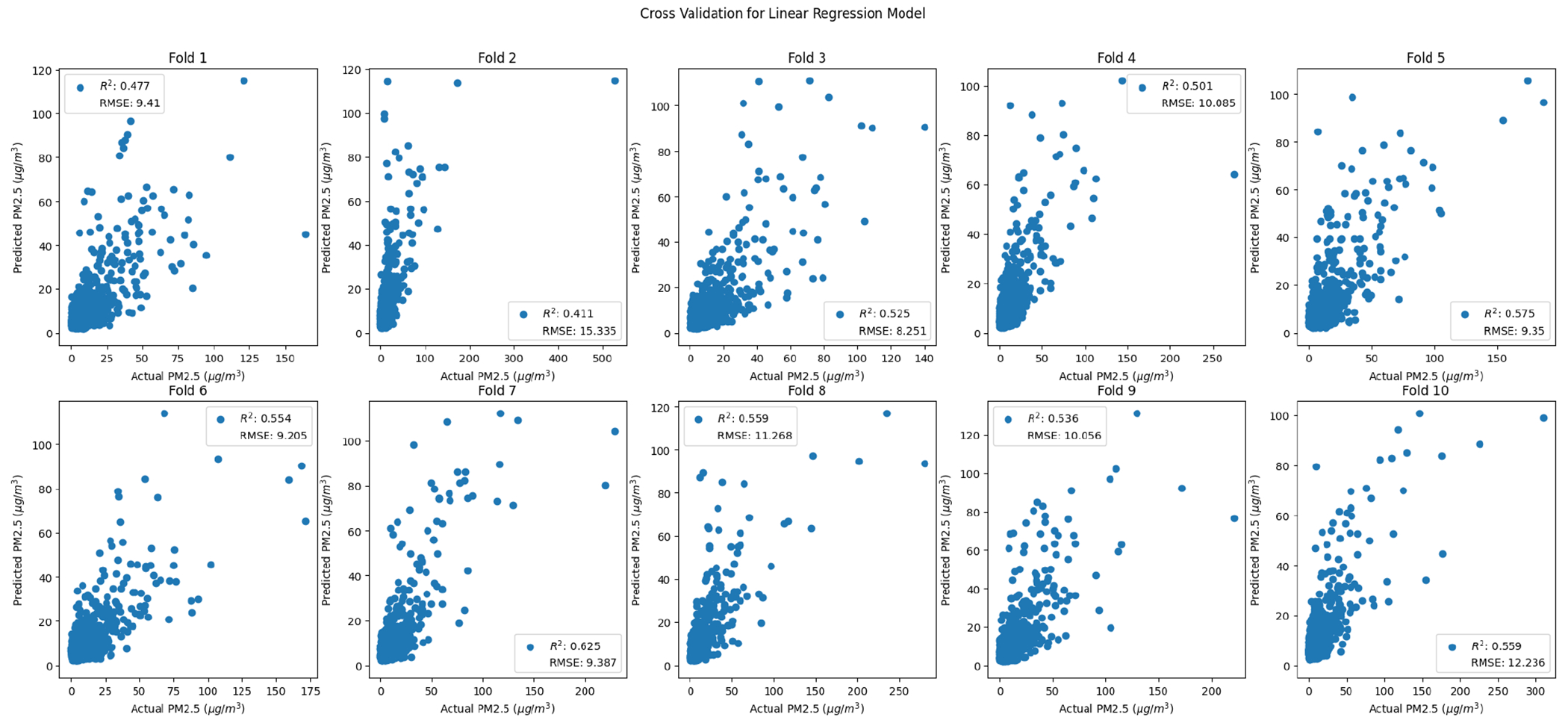
Simple linear regression cross-validation folds.

**Figure 10.**
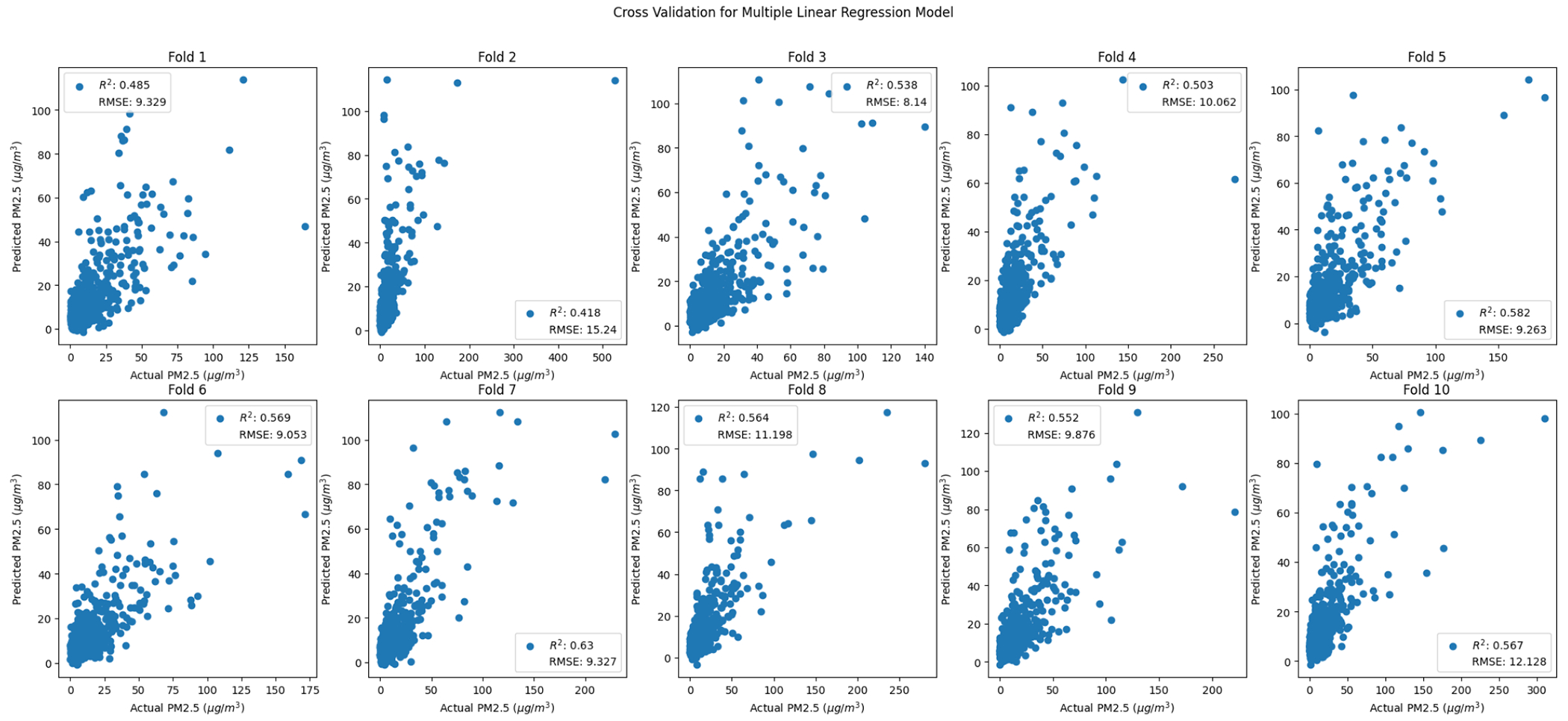
Multiple linear regression cross-validation folds.

As discussed in the methods, 10-fold cross validation was used to measure performance across models (Figures 7–10). For comparison purposes, cross validation was performed on simple-linear-regression and multiple-regression models with the same ten folds used for the random forest cross validation. Based on the cross-validated R^2^ and RMSE values, the random forest method performs better than simple linear and multiple linear regression (Table 7).

Collinearity negatively affects the performance of multiple linear regression models but does not affect the performance of random forest models [42]. Temperature, pressure, and wind direction were the least important factors for multiple linear regression. Removing these variables from the multiple linear regression model improved the RMSE and brought the variance inflation factors of the remaining variables below five. The Pearson correlation coefficients and variance inflation factors are available in Tables A1–A3.

The out-of-bag error estimate uses the data excluded from each bootstrapped sample to provide an additional metric for model generalization [43]. The optimized random forest model generated a 10.53 μg/m^3^ out-of-bag error estimate using the built-in method from SMILE random forest.

There is a built-in method on Google Earth Engine (GEE) for calculating feature importance (Table A4). Due to uncertainty about this built-in method, it was not used for feature engineering. Instead, features were selected based on efficacy in related studies and on their improvement of model metrics.

### 4.3. Montana State-Wide PM2.5 Concentration Map

We published a GEE application that displays daily, PM2.5 values across the map of Montana (https://ee-aspenjkmorgan.projects.earthengine.app/view/mt-hazardous-gas-map, accessed on 18 March 2023, Figure 11). There is a toggle that allows users to select any date from 1 January 2012 to the present. The application combines the AOD, RH, PRES, DPT, WDIR, WIND, and PBLH during the Terra flyover window on that date. If a user requests data from the present day prior to when Terra has passed over Montana, the app displays the values from the day before. The random forest model, with optimized hyperparameters and trained on the cross-validation fold with the lowest RMSE, runs predictions on the satellite data. Users can click anywhere on the map to view the predicted PM2.5 in that location, alongside the coordinates.

**Figure 11.**
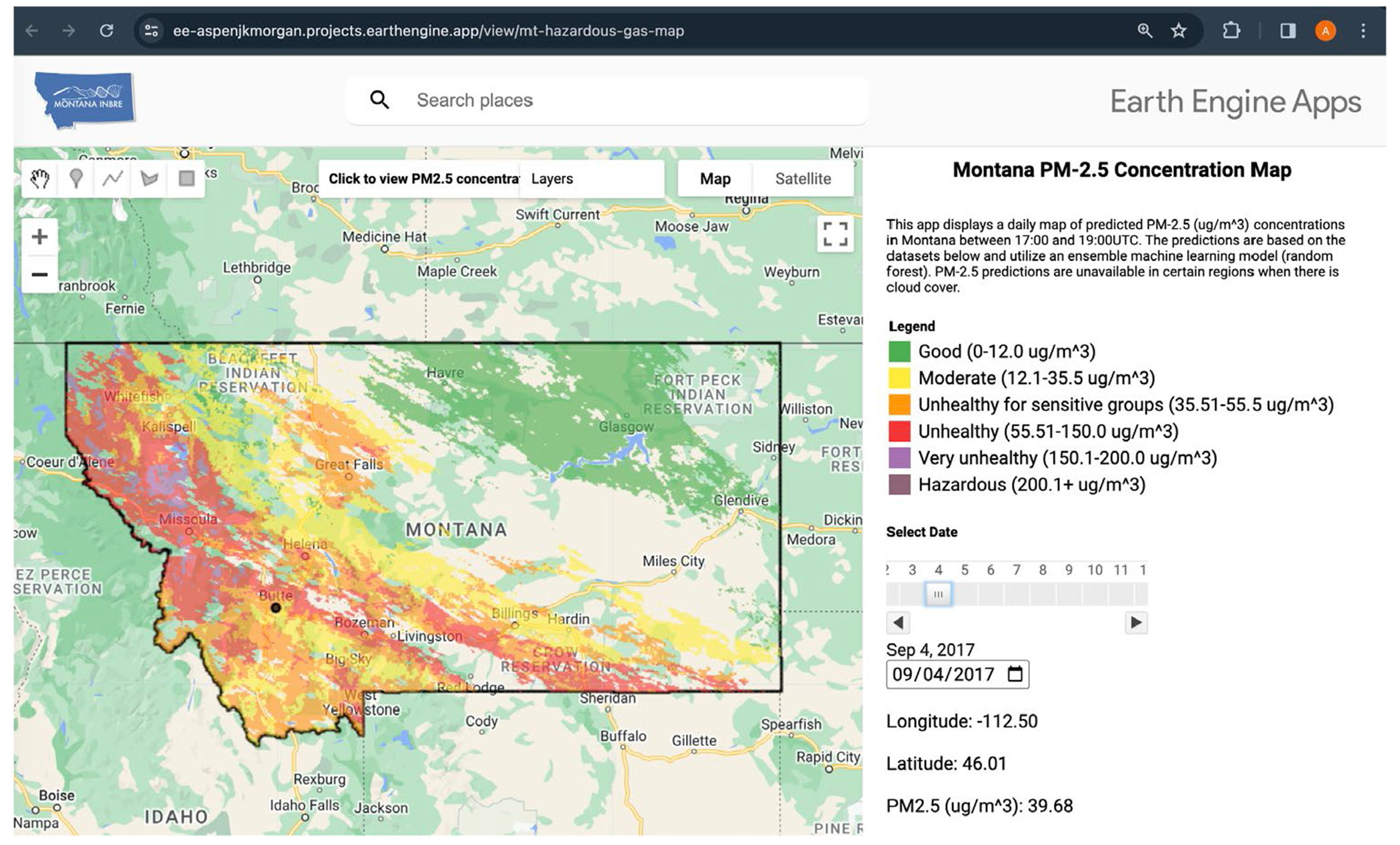
Google Earth Engine App screenshot. Areas without a PM_2.5_ estimate are due to cloud cover or other missing data. PM2.5 data corresponds to daily mean between 17:00 and 19:00 UTC.

The map (Figure 11) displays the predicted PM2.5 values across Montana on 4 September 2017. This date was selected to show the potential of the web app during peak wildfire season. The selected location in Figure 11 corresponds to the Butte ground station at latitude −112.5 and longitude 46.0. During the Terra satellite flyover time on September 4th, 2017, the average PM2.5 value at the Butte ground station was 50.77 μg/m^3^ [33]. The predicted value, 39.68 μg/m^3^, is within reason, given the held-out error of 10.53 μg/m^3^.

## 5. Discussion

### 5.1. Estimating PM2.5 Concentrations Using Ensemble Machine Learning Models

Aerosol optical depth (AOD) has been used to estimate PM2.5 concentrations for several years. Simple linear regression and multiple linear regression have been effective models for predicting PM2.5 in certain regions [17]. However, ensemble models are becoming more popular, particularly in areas where the relationships between PM2.5 and AOD and between PM2.5 and other climate variables are less linear (Table 1). It appears that the random forest model is one of the most common, likely due to its interpretability and relatively small computational burden as compared to other ensemble models like gradient boosting.

There are some drawbacks to ensemble models as compared to linear regression models. For instance, ensemble models perform optimally when their hyperparameters are tuned. This requires grid searching. The most common options are complete grid search (iterating over all hyperparameter combinations) and random grid search (iterating over a random selection of combinations). A complete grid search is more computationally expensive but is guaranteed to find the optimal parameters out of the given options. In general, ensemble models are less widely known, but some can be nearly as interpretable as linear regression. The implementation of an ensemble model is comparable to linear regression but requires some research to understand the underlying mechanics.

### 5.2. Using Google Earth Engine from Beginning to End

Out of the many applications that provide ensemble learning models, GEE offers a relatively limited selection of models and auxiliary functions. For example, there are no built-in methods for cross validation or grid searching. One advantage of GEE is that all computation can be carried out on Google Cloud, which can make a complete grid search over hyperparameters more feasible. Additionally, the ability to create GEE Apps streamlines the process of creating a seamless map of PM2.5 in Montana. By utilizing GEE for modeling, and not just for its datasets, the workflow overall is simplified.

The code for this project (available at https://github.com/mt-pm-concentration-map accessed on 18 March 2023) is open source. The data preparation, cross validation, and final testing were completed in Python using the GEE API and libraries for graphing and searching the data. The cross validation and the held-out testing scripts are organized by model (i.e., simple linear regression, multiple linear regression, and random forest). The JavaScript code used to create the GEE App is also available for reference.

### 5.3. Future Work to Improve the PM2.5 Prediciton Model

**Figure 12.**
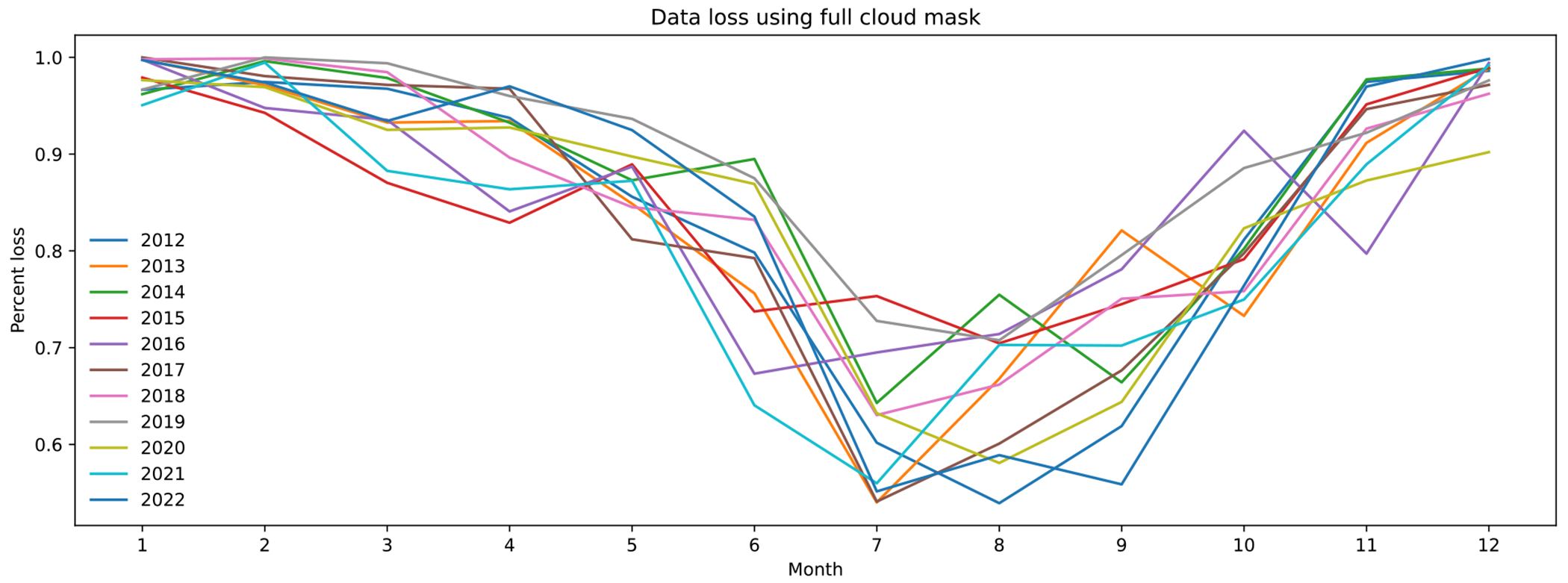
Data loss using full cloud mask.

**Figure 13.**
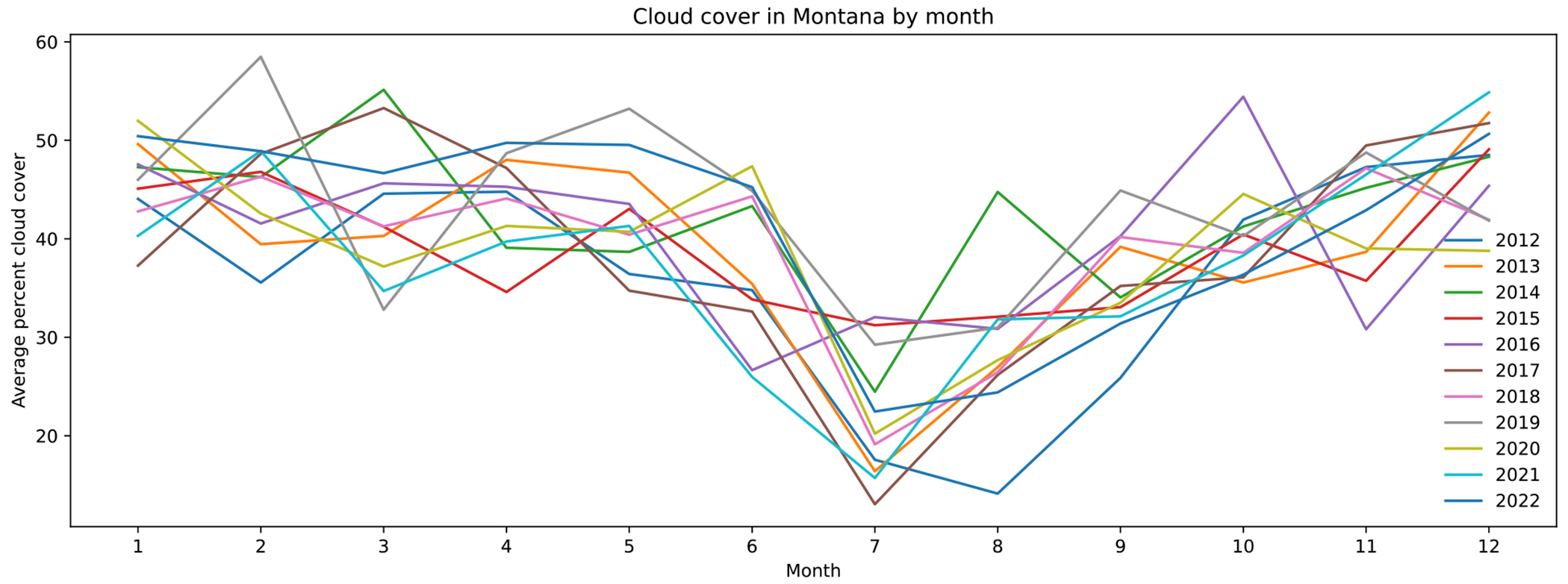
Average percent cloud cover by month.

A major obstacle for predicting PM2.5 in Montana using MCD19A2 was data loss. Over 80 percent of the available ground-station PM2.5 data were lost when combined with MCD19A2 AOD data. Then, an additional five percent was removed by cloud masking to improve the accuracy of the models. One explanation for the data loss is cloud coverage. The data loss in MCD19A2 AOD (Figure 12) follows a similar trend as the average percent cloud coverage in Montana by month (Figure 13).

The studies referenced in Table 1 also utilize MODIS AOD and yet outperform the models used in this study. These other studies either supplemented the MODIS AOD with other AOD data [19], imputed the missing AOD [20,21] or did not use MODIS AOD as the primary input variable in their study [22,23]. As such, filling in the missing AOD data will lead to improvements in both the linear regression and random forest models we trained. There are a variety of methods for imputing or compensating for the missing AOD. Goldberg et al. [42] imputed missing AOD values by using a seasonal average of AOD and adjusting this based on ground-station PM2.5. Hu et al. [19] used the GEOS-Chem model. Brokamp et al. [20] combined two random forest models, one when AOD was available and one when AOD was unavailable. Di et al. [21] used an additional random forest model for imputing AOD with other aerosol satellite data and validated the results using the Aerosol Robotic Network (AERONET). And Yang et al. [22] primarily relied on the top-of-atmosphere reflectance (TOA), which is used to calculate AOD. As there is no daily TOA available on GEE and the GEOS Chem model would substantially increase the project complexity, the methods used by Goldberg et al. [44], Brokamp et al. [20], and Di et al. [21] are promising next steps for improving PM2.5 predictive modeling. And although there are only six AERONET stations in Montana, five of which are in the Missoula area, these stations could be used for partially validating the results of any method of imputing AOD.

#### Evaluating Other Machine Learning Methods and Additional Data Sets

After filling in the missing AOD, we will pursue several other options for improving the accuracy of our PM2.5 predictions. One possibility is incorporating land use variables into our modeling. Several of the studies in Table 1 were primarily focused on PM2.5 from urban pollution and utilized land use variables in their models. All studies in Table 1, besides Hu et al. [19], used the normalized difference vegetation index (NDVI). Brokamp et al. [20] used the length of major roadways, Di et al. [21] used road density, and Hu et al. [19] used population density. These variables were excluded from our modeling for the sake of simplicity and due to our focus on PM2.5 from wildfires rather than urban pollution. However, the effectiveness of these variables for modeling PM2.5 in Montana should be tested. Hu et al. [19] Ghahremanloo et al. [23], and Di et al. [21] also used elevation in their models, and we considered it for this study. However, elevation had a negative impact on all models in preliminary testing.

We also plan to test deep learning models and heterogeneous ensemble machine learning models. Di et al. [45] used a neural network to predict PM2.5 across the United States and achieved a cross-validated R^2^ of 0.84 for their daily predictions at 1 km spatial resolution. Then, they improved their results, achieving a cross-validated R^2^ of 0.86, by stacking a gradient-boosted model, a neural network, and a random forest model in a general additive model [21]. In both [21] and [45], their cross-validated R^2^ was lower in the Western United States, particularly in the Rocky Mountain region, where the cross-validated R^2^ was 0.77 in the 2019 study. Similar machine learning models should be applied to Montana with the optimal variables for this region.

## 6. Conclusions

We used a random forest model to predict daily PM2.5 in Montana at 1km-resolution using aerosol optical depth and other meteorological variables available on Google Earth Engine (GEE). The other meteorological variables—pressure, dewpoint temperature, relative humidity, wind speed, wind direction, and planetary-boundary-layer height—were selected based on their use in other studies and ability to improve the predictive model. The data from 2012 to 2022 were split into 70 percent for training and 30 percent for held-out testing. A GEE random forest model outperformed linear regression on both the cross validation and held-out data. We performed a cross validation grid-search over random forest hyperparameters to optimize the RMSE, resulting in a cross validation R^2^ of 0.572 and RMSE of 9.98 and a held-out R2 and RMSE of 0.487 and 10.53, respectively. We applied the optimized random forest model to generate an interactive PM2.5 map in a GEE web application, available at https://ee-aspenjkmorgan.projects.earthengine.app/view/mt-hazardous-gas-map (accessed on 18 March 2024). The map allows users to select any date from 2012 to 2023 and displays the PM2.5 at any location in Montana. One notable limitation in this study was missing aerosol optical depth from cloud and snow cover. For future improvement, we could estimate the missing AOD using a seasonal average or use an additional machine learning model.

## Figures and Tables

**Table 1.** Summary of random forest literature review.

Citation/Setting/Prediction Model Resolution	Features	Methods	Random Forest CV Metrics
[19] Contiguous USA 2011 Daily at 12 km	Aerosol optical depth Air temperature Dewpoint temperature Visibility Pressure Potential Evaporation Downward longwave-radiation flux Downward shortwave-radiation flux Connective available potential energy Coordinates of ground stations Relative humidity U-wind, V-wind Land use variables * Dummy variables: climate region, day, month Daily 24 h averaged ground-level PM2.5 measurements	Used GEOS-Chem model for imputing missing aerosol optical depth. Convolutional layers (inverse-distance-weighted average function) for nearby PM2.5 measurements and land use variables.	R^2^: 0.80 RMSE: 2.83 μg/m^3^
[20] Cincinnati, USA 2000–2015 Daily at 1 km	Aerosol optical depth (AOD) Visibility Planetary-boundary-layer height Temperature Relative humidity Precipitation Pressure U-wind, V-wind Land use variables * Median PM2.5 from three close days Grid identifier, year, day of year	Removed aerosol optical depth above 1.5, since it indicates rare event for the area. Convolutional layer for nearby PM2.5 values. Combined two random forest models: (1) when AOD was unavailable and (2) when AOD depth was available. Essentially used the missingness of AOD as a predictor.	R^2^: 0.91 RMSE: N/A
[21] Contiguous USA 2000–2015 Daily at 1 km	Aerosol optical depth Surface reflectance Absorbing aerosol index Land use variables * Spatially lagged PM2.5 Latitude Longitude Surface temperature Upward longwave radiation GEOS-Chem PM2.5 estimate	Removed aerosol optical depth over 1.5, based on quality flags. Used random forest to impute AOD using other model variables. Combined gradient boosting, random forest, and neural network in generalized additive model to improve results.	R^2^: 0.73 to 0.901 depending on year RMSE: N/A
[22] Yangtze River Delta, China 2018 Daily at 1 km	Aerosol optical depth Top-of-atmosphere reflectance Planetary-boundary-layer height Surface temperature U-wind, V-wind Relative humidity Pressure Land use variables * Solar zenith and azimuth Sensor zenith and azimuth	Top-of-atmospheric reflectance is the main independent variable, since aerosol optical depth has significant missing data.	R^2^: 0.96 RMSE: 4.21 μg/m^3^
[23] Texas, USA 2014–2018 Daily at 1 km	Aerosol optical depth Air temperature Relative humidity Pressure Wind speed Wind direction Visibility Precipitation Planetary-boundary-layer height Land use variables * Total column densities of dust, sea salt, OC, BC, SO_2_, and SO_4_	Random forest outperformed linear-regression and mixed-effects models.	R^2^: 0.83 to 0.90 depending on year RMSE: N/A

* Land use variables include elevation, NDVI, road network data, forest coverage, impervious surface coverage, etc.

**Table 2.** Datasets.

Dataset	Variable	Units	Spatial Resolution	Temporal Frequency	Available After
Montana DEQ Ground-Stations	PM2.5	μg/m^3^	NA	Hourly	1 January 2012
MCD19A2	OPTICAL DEPTH 047 (AOD)		1 km	Daily	24 February 2000
NOAA NWS RTMA	RH * TMP DPT WIND WDIR PRES	% C C m/s deg Pa	2.5 km	Hourly	1 January 2011
M2T1NXFLX	PBLH	m	(70, 55) km	Hourly	1 January 1980

* RH was calculated using DPT and TMP [34]. MCD19A2 is from the NASA MODIS satellite and M2T1NXFLX is from the NASA MERRA-2 reanalysis.

**Table 3.** Data loss by variable.

Variable	PM25	AOD	Cloud Mask	DPT, PRES, RH	WIND	WDIR
Observations	123,932	31,511	19,151	18,950	18,943	18,800

**Table 4.** Cross validation with and without cloud masking.

	Cross Validated R^2^		
	Simple Linear Regression	Multiple Linear Regression	Random Forest
Cloud Mask	0.532	0.541	0.572
No Cloud Mask	0.485	0.498	0.557

**Table 5.** Grid search space.

Parameter	Range	Optimal Value
Bag fraction	[0.1, 0.2, …, 0.9]	0.9
Minimum leaf value	[1, 2, …, 10]	3
Variables per split	[1, 2, …, 7]	3

**Table 6.** Montana PM2.5 concentration data summary for 2017. Please note that the lower bound is inclusive and upper bound is exclusive in each category range.

Station Name	Good [0.0–12.0)	Moderate [12.0–35.5)	Unhealthy for Sensitive Groups [35.5–55.5)	Unhealthy [55.5–150.5)	Very Unhealthy [150.5–250.5)	Hazardous [250.5–500.0)	[500.0–1000.0)	Total Measurements *
Billings Lockwood	665	55	3	0	0	0	1	753
Bozeman	6062	1514	160	29	0	0	0	8760
Broadus	5737	1578	256	147	2	0	0	8760
Butte	4951	2170	462	167	7	0	0	8760
Flathead Valley	5996	1606	163	101	67	2	0	8760
Frenchtown	6010	2106	260	215	15	8	0	8760
Great Falls	6854	1256	92	19	0	0	0	8760
Hamilton	5562	1863	518	395	10	0	0	8760
Helena	5316	1999	459	277	0	0	0	8760
Lewistown	6129	993	125	63	0	0	0	8760
Libby	4689	3078	359	135	30	0	0	8760
Malta	6292	923	64	33	0	0	0	8760
Missoula	5997	1714	254	283	20	4	0	8760
NCore	6384	709	194	68	6	0	0	8760
Seeley Lake	3628	2954	779	594	96	181	193	8760
Sidney	4539	751	51	35	0	0	0	5969
Thompson Falls	2841	1409	156	67	72	40	1	4682
West Yellowstone	6152	767	67	15	2	1	2	8755

* Please note that total measurements include all the reported values, including invalid values (negative values).

**Table 7.** Validation results.

Type of Validation	R^2^	RMSE (μg/m^3^)
	Simple Linear Regression	
Cross Validation	0.532	10.46
Held-out Data	0.440	11.16
	Multiple Regression	
Cross Validation	0.541	10.36
Held-out Data	0.448	11.07
	Random Forest	
Cross Validation	0.572	9.98
Held-out data	0.487	10.53

## Data Availability

Data is available upon request from the corresponding author.

## Appendix A. Supplementary Data

**Table A1.** Pearson correlation coefficients among independent variables.

Feature	AOD	DPT	RH	WIND	WDIR	PRES	TMP	PBLH
AOD	1							
DPT	0.13	1						
RH	−0.08	0.09	1					
WIND	−0.08	−0.21	−0.19	1				
WDIR	−0.03	−0.13	−0.15	0.21	1			
PRES	−0.02	0.15	0.18	0.03	−0.10	1		
TMP	0.17	0.70	−0.63	−0.03	0.01	−0.01	1	
PBLH	0.05	0.35	−0.52	0.015	0.05	−0.17	0.64	1

**Table A2.** Variance inflation factor for random forest variables.

Feature	VIF
DPT	1.95
PBLH	13.79
PRES	44.59
RH	13.30
WDIR	6.74
WIND	3.04
AOD	1.55

**Table A3.** Variance inflation factor for multiple-linear-regression variables.

Feature	VIF
AOD	1.49
WIND	2.62
RH	3.39
DPT	1.82
PBLH	4.88

**Table A4.** Feature importance.

Variable	Relative Importance
AOD—Aerosol Optical Depth	29.6%
DPT—Dewpoint	14.5%
RH—Relative Humidity	12.9%
WIND—Wind Speed	12.4%
PRES—Pressure	11.1%
PBLH—Planetary Boundary Height	10.9%
WDIR—Wind Direction	8.5%
